# Parsonage–Turner Syndrome and Vocal Fold Paresis Following Soberana 2 FINLAY‐FR‐2 COVID‐19 Vaccination: A Case Report

**DOI:** 10.1002/ccr3.71348

**Published:** 2025-10-31

**Authors:** Amir‐Hassan Bordbari, Negar Heidari, Narges Karimi

**Affiliations:** ^1^ Student Research Committee, School of Medicine Mazandaran University of Medical Sciences Sari Iran; ^2^ Department of Neurology, Clinical Research Development Unit of Bou Ali Sina Hospital Mazandaran University of Medical Sciences Sari Iran; ^3^ Department of Neurology, School of Medicine, Immunogenetics Research Center, Clinical Research Development Unit of Bou Ali Sina Hospital Mazandaran University of Medical Sciences Sari Iran

**Keywords:** brachial plexus neuritis, COVID‐19 vaccines, Parsonage–Turner syndrome, vocal fold paralysis

## Abstract

This case report presents a 58‐year‐old male who developed Parsonage–Turner syndrome (PTS) and vocal cord paresis after receiving the Soberana 2 FINLAY‐FR‐2 COVID‐19 vaccine. The patient experienced escalating pain and weakness in both shoulders and upper limbs after each vaccine dose, with subsequent neurological symptoms including headaches, dysphagia, hoarseness, numbness, dyspnea, and chest discomfort. Neurological examinations and electrodiagnostic studies confirmed upper trunk brachial plexopathy along with vocal fold paresis. This patient was treated with intravenous methylprednisolone. Despite therapeutic intervention, the patient exhibited persistent neurological deficits during follow‐up.

**Summary**

- Parsons–Turner syndrome and vocal cord paralysis may occur after vaccination.
- In this report, we present a patient with shoulder pain and weakness and voice disturbance after receiving the COVID‐19 vaccine.

## 1 Introduction

Parsonage–Turner syndrome (PTS), also known as neuralgic amyotrophy (NA) and brachial plexus neuritis, is a rare neurological syndrome characterized by the sudden onset of severe unilateral pain and sensory disturbance followed by muscle weakness in the shoulder girdle and proximal upper limb [1, 2]. The syndrome happens due to damage to the brachial plexus. Medical Research Council (MRC) Grading is often used for the examination of muscle stretch, with grading 0 (no contraction) to 5 (normal). With MRC, the strength of each muscle is assessed individually based on muscle function in brachial plexus injury patients. Typically, the condition initiates with sudden and persistent pain in the shoulder girdle and upper limb [2]. The pain is usually not positional, worsens during the night, which may disrupt sleep, and often does not respond to common analgesics [2, 3]. While recovery may occur within 1–2 weeks for some patients, others experience prolonged pain. Motor weakness develops several days to weeks after the onset of pain, while sensory impairments are not always present. Atrophy becomes evident after about a month [2]. Atypical forms of PTS are associated with simultaneous or individual involvement of other peripheral nerves such as the median, radial, and phrenic nerves [3, 4]. The overall incidence of this syndrome is 1.64 per 100,000 individuals, and cases have been reported in individuals aged 3 months to 75 years [2]. Various risk factors have been reported for this condition, such as viral infections, vaccination, trauma, and surgery [1, 2]. With the onset of the coronavirus disease 2019 (COVID‐19) pandemic, numerous cases of PTS following COVID‐19 infection and vaccination have been reported [1]. Several studies reported neurological disorders among people who have received the COVID‐19 vaccines, including vascular, immune, and infectious disorders [5, 6, 7]. Cranial and peripheral neuropathy after injection of COVID‐19 vaccination were reported. It included Bell's palsy, diminished vision, olfactory impairment, hearing loss, Guillain–Barre syndrome (GBS), and small fiber neuropathy [7]. The mechanisms underlying these neurological effects are not yet fully understood, but it is hypothesized that immune‐mediated processes or direct effects of vaccine components on the nervous system may play a role. It is crucial for healthcare professionals to remain aware of these potential neurological complications, as timely recognition and intervention can significantly influence the outcome of patients. One of the disorders reported following the COVID‐19 infection was vocal fold paresis (VFP), which occurs secondary to injury to one or both of the recurrent laryngeal nerves. Some literature has reported VFP following COVID‐19 vaccination [8, 9]. Moreover, VFP and other cranial nerve‐related issues have occurred as potential complications following vaccination, although these manifestations are remarkably rare. Initial trials on the safety and efficacy of the Soberana 2 FINLAY‐FR‐2 COVID‐19 vaccination in Cuba and Iran have not reported significant neurological complications [10, 11]. In this report, we describe a patient who developed PTS and VFP after receiving the Soberana COVID‐19 vaccine. As far as we know, this is the first reported case of PTS associated with the Soberana 2 COVID‐19 vaccine.

## 2 Case Presentation/Examination

Our patient is a 58‐year‐old right‐handed man who developed pain and weakness in the left shoulder and upper limb 1 week after receiving the first dose of the Soberana 2 FINLAY‐FR‐2 COVID‐19 vaccine in the left deltoid. Despite the symptoms, the patient received the second shot in the right deltoid 4 weeks later. After the second shot, similar symptoms developed on the right side. These symptoms did not get any better, and the patient refused to seek medical help.

Approximately 8 weeks after receiving the second dose, the patient experienced sudden onset of headache, intensifying pain and weakness in both shoulders and upper limbs, with the right arm being more affected, along with right cervical pain, dyspnea, and chest discomfort, hoarseness, dysphagia, and numbness in the right deltoid and left forearm. The patient had no past medical history including trauma, infections, or diabetes, and any family history was denied.

About 2 months later, the patient was referred to our hospital, and he was admitted with prior symptoms. Upon admission, vital signs and general examinations were unremarkable. On neurologic examination, atrophy was noted in the right deltoid and left biceps muscles. Weakness of right shoulder abduction and left elbow flexion were detected. Medical Research Council (MRC) score of the right deltoid muscle was 0/5, and the left deltoid muscle had an MRC score of 4+/5. Right and left biceps muscles had an MRC score of 4+/5 and 3/5, respectively, while both triceps had full power. The right flexor digitorum profundus (FDP) had full power, but the left FDP had an MRC score of 4−/5. Hypoesthesia was documented in the right deltoid region and left lateral forearm. Deep tendon reflexes were muted except for both triceps, which were 2+.

## 3 Differential Diagnosis, Investigations, and Treatment

Differential diagnosis in patients presenting with severe, unilateral shoulder pain includes cervical disc herniation or intervertebral foramen stenosis causing cervical radiculopathy or mass lesions compressing the brachial plexus or individual nerves. Brain computerized tomography (CT) scan, brain magnetic resonance imaging (MRI), and brachial MRI were normal. However, cervical MRI showed protrusion at C3–C4 and C5–C6 without canal narrowing. Thoracic MRI showed central protrusion at T7–T8 and T8–T9, and thecal sac indentation in T12–L1. A laryngoscopy was performed due to hoarseness, which confirmed weak motility in the left vocal cord. The laboratory examination results were as follows: serum glucose 131 mg/dL; blood urea nitrogen: 50 mg/dL; creatinine 1.3 mg/dL; alanine aminotransferase 13 IU/L; aspartate aminotransferase 23 IU/L; sodium 135 mmol/L; potassium 3.9 mmol/L; white blood cell count 6700 cells per microliter (neutrophils = 43%; lymphocytes = 54%); erythrocyte sedimentation rate 32 mm/h, hemoglobin 13.6 g/dL. Rheumatological blood tests, virology blood test including HIV, HBV, and HCV, and CSF analysis were normal. Electromyography and nerve conduction velocity (EMG‐NCV) revealed decreased CMAP amplitude in right axillary and left musculocutaneous nerves with fibrillations, positive sharp waves, and neurogenic pattern in left biceps and right deltoid muscles (Table 1).

**TABLE 1:** Electrodiagnostic study.

Nerves	Latency (ms)		Amplitude (mV)		Conduction velocity (m/s)	
	Right	Left	Right	Left	Right	Left
Median (S)	3.2	2.2	20.5	23.4	47	57
Ulanr (S)	2.6	2.5	19.3	18.4	48	49
Median (M)						
Wrist	4.0	3.9	6.5	6.0		
Elbow	10.5	9.5	6.4	5.9	49	49
Ulnar (M)						
Wrist	2.5	2.5	9.7	9.9		
B. Elbow	7.1	6.8	9.0	9.8	50	55
A. Elbow	8.3	7.9	8.9	9.4	52	58
Axillary (M)	2.6	3.6	**2.8**	11.1		
Musculocutaneous (M)	4.7	4.3	8.9	**3.1**		

*Note:* Bold values show decreased amplitude in right axillart and left musculocutaneous nerves.

Abbreviations: A, above; B, blow; M, motor; S, sensory.

The patient was treated with intravenous methylprednisolone 1 g daily, totaling 3 g, leading to the alleviation of dysphagia. Upon discharge, the patient was prescribed oral prednisolone.

## 4 Outcome and Follow‐Up

Three months later, there was no improvement in the patient's neurologic deficits during a follow‐up visit. Unfortunately, the patient declined further therapy and follow‐up.

## 5 Discussion

In this case report, we present a 58‐year‐old patient who developed symptoms of Parsonage–Turner syndrome (PTS) after receiving each dose of the Soberana vaccine. Two months following the second dose, the patient experienced a sudden worsening of previous symptoms and the onset of sensory disturbances in the upper limbs, severe headaches, voice hoarseness, dysphagia, dyspnea, and chest discomfort. Despite seeking medical attention later than advisable, electromyography and nerve conduction velocity (EMG‐NCV) tests confirmed upper trunk involvement and validated a diagnosis of PTS. PTS often goes undiagnosed or misdiagnosed due to its varied and complex clinical presentation [12]. When these symptoms arise post‐vaccination or following a COVID‐19 infection, it is essential to consider PTS as a possible diagnosis [1]. A thorough patient history and comprehensive physical examination are critical initial diagnostic steps. MRI and EMG‐NCV can be valuable tools for confirming the diagnosis while excluding other potential causes [1, 13]. Differential diagnoses include conditions such as rotator cuff tear, adhesive capsulitis, cervical radiculopathy, amyotrophic lateral sclerosis (ALS), and peripheral nerve lesions [14].

Viral infections and vaccinations are the common risk factors for PTS [2]. PTS has been reported after various vaccines [1, 15]. A systematic review by Ameer et al. explored the correlation between both COVID‐19 infection and vaccination and PTS, revealing a higher occurrence among middle‐aged men [1]. Furthermore, PTS associated with COVID‐19 vaccination tends to manifest earlier than cases resulting from actual COVID‐19 infection. Additionally, COVID‐19 vaccines are more likely to affect both the upper and lower trunks of the brachial plexus, whereas the infection primarily influences only the upper trunk [1]. Lakkireddy et al. reported a patient with PTS 5 weeks following Covishield (AstraZeneca ChAdOx1 nCoV‐19) COVID‐19 vaccination [16]. Electrodiagnostic study shows decreased motor amplitude in the right axillary and musculocutaneous nerves [16]. The patient was treated with 1 g methylprednisolone intravenously for 3 days followed by oral steroids for 3 weeks [16]. Rosca et al. described 56 cases of PTS after the administration of mRNA and viral vector COVID‐19 vaccines in a systematic review, and improvement was more in the viral vector than in the mRNA vaccine group [17]. Queler et al. described two cases of PTS 13 h and 18 days following the Pfizer‐BioNTech BNT162b2 and Moderna mRNA‐1273 COVID‐19 vaccinations [18]. The patients had weakness in wrist flexion and forearm pronation [18]. The diagnosis of PTS was documented via both electrodiagnostic testing and 3.0‐T MR neurography [18]. The mRNA vaccines provoke potent type I interferon reactions, which promote inflammation and may be associated with an increased risk of autoimmune reactions [18]. Shield et al. reported six PTS patients after Pfizer‐BioNTech (four patients) and Moderna COVID‐19 (two patients) vaccinations [19]. After treatment with prednisolone, three out of six patients did not show improvement in muscle weakness, whereas three other patients had some recovery in muscle force [19].

Although the exact mechanism is not fully understood, it has been suggested that the involvement of the brachial plexus may be due to either direct viral infection or an immune response [1, 2]. Chakrabarti et al. reported two possibilities in the patient who rapidly progressed to dementia following ChAdOx1 nCoV‐19 vaccination: a direct toxicity of the S protein or the toxicity of anti‐S protein antibodies [20]. Protein S induces a strong adaptive immune response by various types of activated B and T lymphocytes [20]. The vaccine induces molecular mimicry due to the similarity between the epitopes in the vaccine and myelin and axonal glycoproteins [21]. It is unlikely that PTS is secondary to direct nerve injury from vaccination, as other peripheral neuropathies and PTS contralateral to the vaccine injection side are frequently reported [18]. Besides, the inflammatory nature of the disease has been confirmed by MRI findings that reveal inflammation in the nerves and muscles [1]. Additionally, reports of PTS occurring in family members suggest a possible genetic component [1, 13]. There is no definitive cure for PTS, so treatment focuses on managing symptoms and improving function while allowing time for potential nerve recovery. The main goal of treatment is to retain as much strength and mobility in the affected arm and shoulder as possible. Treatment options for PTS include steroids, pregabalin, physical therapy, and bracing [1]. While clinical trials are lacking, evidence suggests the potential benefits of initial oral steroids [13, 22] and supportive data suggest a positive effect of Intravenous Immunoglobulin (IVIG) therapy [23, 24]. Corticosteroids and intravenous immunoglobulin have been revealed to improve pain in the acute stage. Nevertheless, these medications have not been as effective after the first 2 weeks [25]. The severity of clinical symptoms of PTS depends on the severity and extent of the nerve damage, and about 80% of patients may recover with rehabilitation within 2–3 years [13]. In cases of COVID‐related PTS, most patients achieve partial recovery in less than a year [1]. Reinnervation—a process involving nerve fiber regrowth—is known to begin around 6–12 months after disease onset due to gradual axonal regeneration [2]. Surgical therapy, including neurolysis or nerve transfer, may be performed in patients who have persistent symptoms. In 90% of the patients, neurolysis improves symptoms [26].

Beyond PTS symptoms, this report highlights other neurological complications associated with the COVID‐19 vaccine. Notably, cases of vocal fold paresis and paralysis (VFP) have been reported following various vaccinations, including those for COVID‐19 [8, 9]. Hamdi et al. documented 20 cases of VFP after receiving Pfizer‐BioNTech, Moderna, and Janssen COVID‐19 vaccinations, all of whom were confirmed through laryngoscopy [8]. Jama et al. described a patient who developed unilateral VFP after the first dose of the Oxford‐AstraZeneca ChAdOx1 nCov‐19 vaccine [27]. Lehrer et al. described two cases of unilateral VFP after administration of the BNT162b2 vaccine [28]. Son et al. reported an 82‐year‐old female patient with dyspnea and stridor diagnosed with bilateral VFP 3 days after the third COVID‐19 mRNA vaccination (Comirnaty, Pfizer‐BioNTech, USA) [29]. To the best of our knowledge, we are reporting the first case of vocal fold paresis associated with the Soberana COVID‐19 vaccine. This unilateral involvement began suddenly with other symptoms approximately 2 months after the second shot and was confirmed through laryngoscopy. It is uncertain whether this issue is connected to PTS. Furthermore, additional studies are needed to investigate the relationship between VFP and the COVID‐19 vaccine. Involvement of the phrenic nerve and diaphragm dysfunction are among the mononeuropathies that have been reported in association with PTS [1, 4]. However, this co‐occurrence is rare; an observational study indicated that only 7.6% of PTS patients had phrenic nerve involvement [4, 30]. In this case report, considering the patient's medical history, the possibility of phrenic nerve involvement is also raised. However, further investigations were not conducted, and therefore, the certainty of this matter cannot be confirmed.

## 6 Conclusion

Herein, we described a patient with PTS and vocal fold paresis after receiving the COVID‐19 Soberana vaccine. However, PTS and VFP are rarely mentioned in the literature as prominent side effects of vaccination and are largely outweighed by the benefits of vaccination. The link between the COVID‐19 vaccine and PTS is not well understood, but it is likely an abnormal reaction of the immune system after vaccination. VFP could be caused by damage to the recurrent laryngeal nerve, which could be part of an immune‐ and viral‐like mediated inflammatory reaction to the vaccine. Finally, further studies and research, including cellular and molecular studies, are needed to further investigate this disorder, which may be accidental or due to vaccination.

## Author Contributions

**Amir‐Hassan Bordbari:** conceptualization, data curation, formal analysis, resources, visualization, writing – original draft. **Negar Heidari:** conceptualization, data curation, investigation, resources, writing – original draft. **Narges Karimi:** conceptualization, data curation, formal analysis, investigation, project administration, supervision, validation, visualization, writing – review and editing.

## Consent

The authors confirmed that the signed consent was obtained from the patient in accordance with the journal's patient consent policy.

## Conflicts of Interest

The authors declare no conflicts of interest.

## Data Availability

Data sharing not applicable to this article as no datasets were generated or analyzed during the current study. All data have described in the manuscript.
